# Inflammatory Pseudotumor of the Head Presenting with Hemiparesis and Aphasia

**DOI:** 10.1155/2011/176546

**Published:** 2011-07-14

**Authors:** K. Saifudheen, James Jose, V. Abdul Gafoor

**Affiliations:** Department of Neurology, Medical College, Calicut 8, Kerala 673008, India

## Abstract

Inflammatory pseudotumor most commonly occurs in the orbit and produces orbital pseudotumor, but extension into brain parenchyma is uncommon. We report a case of inflammatory pseudotumor involving sphenoid sinus, cavernous sinus, superior orbital fissure, orbital muscle, and intracranial extension into left temporal lobe producing right hemiparesis and wernicke's aphasia. The patient improved clinically and radiologically with steroid administration. This paper provides an insight into the spectrum of involvement of inflammatory pseudotumor and the importance of early diagnosis of the benign condition.

## 1. Introduction

Inflammatory pseudotumor (IPT) is a nonneoplastic inflammatory process that characterized histologically by the presence of acute and chronic inflammatory cells with a variable fibrous response [1]. IPT most commonly involves the orbit and causes orbital pseudotumor, but extension into intracranial and extracranial areas are rarely reported [2]. The condition can mimic invasive tumors both clinically and radiologically. In a confirmed cases of IPT, most cases show good improvement with steroid therapy. 

## 2. Case Report

 A 50-year-old man was admitted with progressive right-sided hemiparesis and aphasia of 5 days duration. The patient was a diabetic for the last 4 years on oral hypoglycemic drug with good glycemic control. His symptom started as painful ophthalmoplegia in January 2008. He had involvement of 3rd, 4th, and 6th nerve on the left eye and 3rd and 4th nerve on the right eye. His visual acuity was 6/60 in the left eye and 6/36 in the right. Optic fundi were normal. Pupils were normal. Sensory loss was noted over left maxillary nerve distribution.

**Figure 1:**
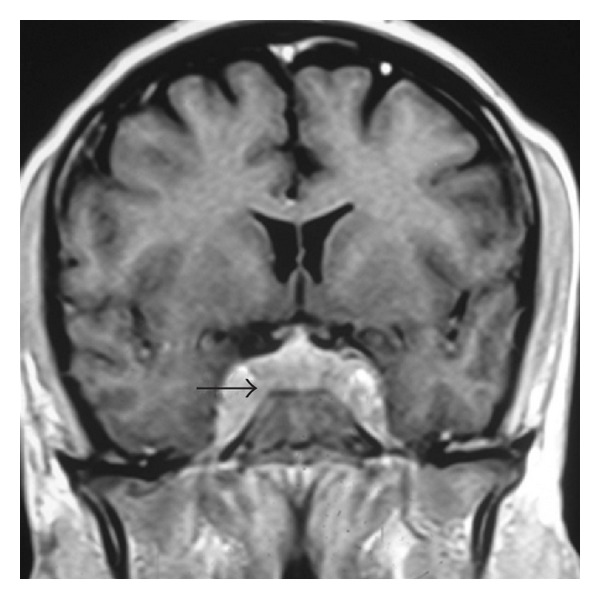
Coronal T1-weighted contrast image shows enhancing lesion in the cavernous sinus bilaterally with sphenoid sinus fullness (black arrow).

 MRI of brain demonstrated (Figure 1) an enhancing lesion in the cavernous sinus bilaterally with sphenoid sinus fullness. Sphenoid mucosal biopsy demonstrated inflammatory tissue with aggregate of lymphoid cell. Patient received a course of oral steroid (prednisolone 40 mg) for 5 days and was lost at followup. 

**Figure 2:**
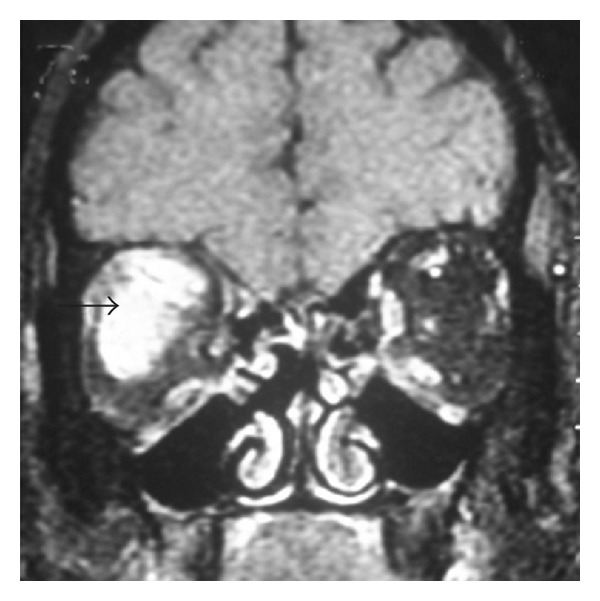
Coronal T1-weighted contrast image shows proptosis of the right globe. There is intense enhancement and enlargement of the right superior and lateral recti (black arrow).

 In September 2008, he was admitted in another center with severe chemosis and proptosis of right eye and reappearance of diplopia. Examination showed partial 3rd and 6th nerve palsy on the right eye. The left eye was normal. MRI of brain and orbital area showed proptosis of right globe. There was enlargement of the right superior and the lateral recti from the retrobulbar region up to the orbital apex (Figure 2). Lesion appears hypointense on T1 and hyperintense on T2 WI and FLAIR. On contrast scan, there is intense enhancement of the lesion, extending from the orbit through the superior orbital fissure into anterior cavernous sinus. In the left orbit, the medial rectus showed mild enlargement with contrast enhancement. Patient received oral steroid (prednisolone 40 mg) for 1 week with substantial improvement of his chemosis, proptosis, and diplopia.

**Figure 3:**
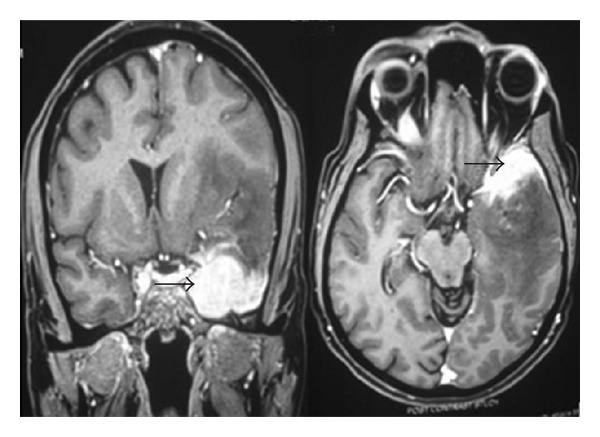
Coronal and Axial T1-weighted contrast image shows intensely enhancing extra-axial mass lesion extending from left cavernous area compressing and invading left anterior temporal lobe (black arrow). There is significant perilesional edema. Lateral ventricle is compressed with midline shift to the right.

**Figure 4:**
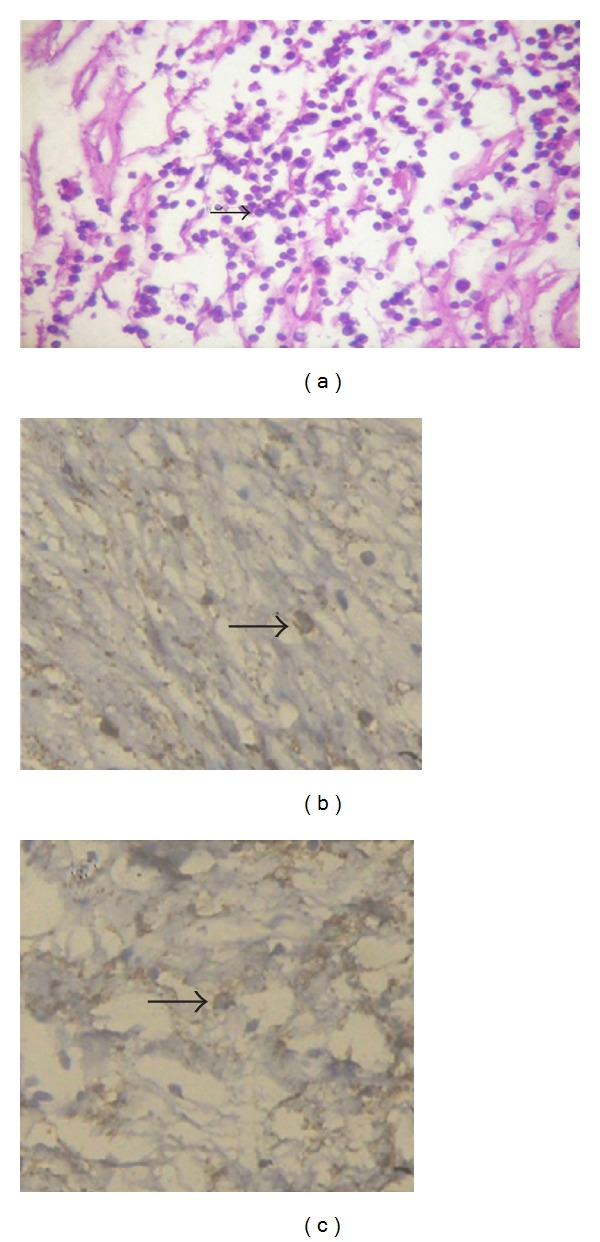
(a) Histopathology showing mature lymphocyte infiltration with occasional macrophage; (b) and (c) immunohistochemical staining for CD3 and CD20 showing the CD3 and CD20 positive lymphocyte.

In February 2009, the patient readmitted in our neurology department because of progressive right-sided hemiparesis, aphasia, and headache. There was no proptosis, visual symptom, squint, or extraocular paresis. MRI, brain showed (Figure 3) intensely enhancing extra-axial mass lesion extending from the left cavernous area compressing and invading the left anterior temporal lobe. An area of hemorrhage was noted in the periphery of lesion. There was significant perilesional edema. The lateral ventricle is compressed with midline shift to the right. Orbital globe, intraorbital muscle are normal. Laboratory workup showed normal hemogram and erythrocyte sedimentation rate. His serum bilirubin, urea, creatinine, and glucose levels were respectively 0.8 mg/dL (normal: 0.3–1.3 mg/dL), 24 mg/dL (normal: 10–50 mg/dL, 0.8 mg/dL (normal: 0.3–1.4 mg/dL), and 100 mg/dL (normal: 90–110 mg/dL). Peripheral smear was normal. Thyroid profile and serum cortisol were within normal limits. VDRL was nonreactive. HIV serology, HBsAg, and HCVAb in the blood were negative. Immunological tests (rheumatoid factor, antinuclear antibodies, cANCA, pANCA, SSA, SSB antibodies, and angiotensin-converting enzyme) were normal. Examination of the cerebrospinal fluid revealed 4 lymphocytes, Sugar: 56, and protein: 35 mg, and polymerase chain reaction for tuberculosis was negative. Biopsy specimens from left anterior temporal and extratemporal mass lesion showed aggregates of mature lymphocyte infiltration with occasional macrophage. There were no abnormal cell and histiocyte. Immunohistochemical staining for CD3 and CD20 showed that the lymphocytes were positive for both CD3 and CD20 (Figure 4). Fungal, AFB stains, and culture of biopsy were negative. Histopathology was consistent with IPT. 

**Figure 5:**
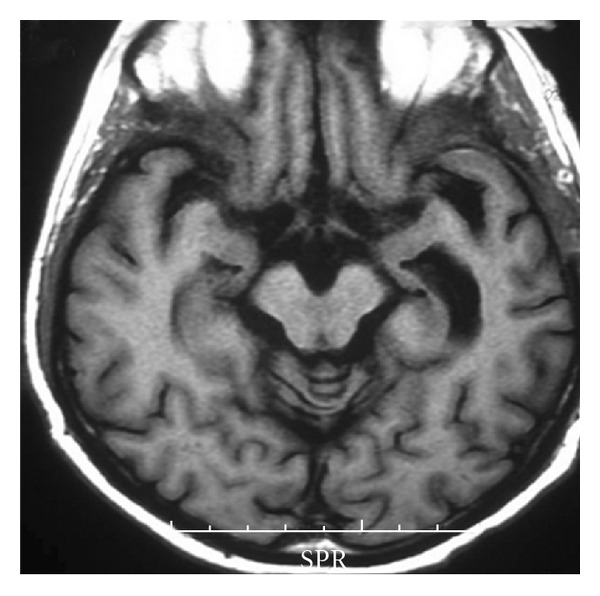
Axial T1-weighted contrast image shows almost complete resolution of the temporal lobe lesion with mild dilatation of the left temporal horn.

 As the patient did not receive a full course of steroid at any time during his illness, the patient was started on high-dose parenteral steroid (Dexamethasone, 12 mg/d). After 1 week, it was changed to oral prednisone (1 mg/kg) for the next one month and then gradually tapered over next 3 months. His glycemic status was controlled with insulin. There was rapid improvement of the symptoms within the first week of treatment. A follow-up MRI study, 1 month later, showed almost complete reduction of the lesion with mild dilatation of the left temporal horn (Figure 5). He was asymptomatic on the last followup after one year. 

## 3. Discussion

IPT is a rare chronic inflammatory tumefaction of unknown origin. The condition was first described in 1905 by Birch-Hirschfield in their orbital localization [2] and was so named by Umiker et al. in 1954 because of its propensity to clinically and radiologically mimic a malignant process [3]. 

 IPT of the Head and neck can affect the orbit (orbital pseudotumor) [4], orbital apex (orbital apex syndrome), superior orbital fissure (superior orbital fissure syndrome) [5], and anterior, middle, and posterior cavernous sinus (cavernous syndrome) [4]. Other extraorbital sites include nasal cavity [3], nasopharynx [2], maxillary sinus [2], sphenoid sinus [6], infratemporal fossa [4], choroid plexus [5], larynx and trachea [3], and skull bone [3]. Extension into brain parenchyma is rarely reported. In a series of 90 consecutive biopsy-proven cases of orbital pseudotumor, eight cases (8.9%) showed radiologic evidence of intracranial spread [6], but invasion into temporal lobe producing hemiparesis and wernicke's aphasia are not reported in the literature. There is some speculation in the literature that IPT, Tolosa-Hunt syndrome, and idiopathic hypertrophic pachymeningitis seem to be part of inflammatory disorders that have diverse locations but share similar histologic, clinical, and imaging findings [7].

 The differential diagnosis for our patient's clinical picture include malignant diseases (lymphoma, leukemias, rhabdomyosarcoma, Ewing's sarcoma, and primitive neuroectodermal tumors and lymphomas), sarcoidosis, Wegener's granulomatosis, vasculitis, tuberculosis, and fungal infections, such as aspergillosis and mucormycosis, are among the possibilities. These conditions are promptly excluded as a likely cause by extensive workup in our case.

A definite diagnosis of IPT is made by the biopsy. Histopathology shows a nonspecific infiltrate of inflammatory cells composed of lymphocytes, plasma cells, neutrophils, and macrophages. Depending on the chronicity, varying degrees of fibrosis may be seen. Immunohistochemical studies of T- and B-cell subpopulations are helpful in distinguishing IPT from lymphoma. IPT usually contain both T cells and B cells, whereas in lymphoma, (clonal) B- or T-cell populations predominate [3]. Histopathology of temporal lobe mass lesion in our patient was positive for both CD3 and CD20. Treatment consists of high-dose oral corticosteroids. Most cases show improvement within 48–72 hours. There is no consensus regarding the duration of treatment. We have to individualize depending on the severity of disease and follow-up imaging. Low-dose radiation therapy may be considered for cases in which the use of steroids is contraindicated, or there is a poor response to steroids. Surgical excision is rarely indicated. Our patient responded adequately to steroid treatment. An analysis of our case illustrates the extensive nature and the diverse presentation of IPT, involving cavernous sinus, sphenoid sinus, the superior orbital fissure, orbital muscle, and intracranial spread into temporal lobe. 

 Knowledge of the spectrum of involvement of IPT can help in early diagnosis and avoid unnecessary radical surgery. However, a definitive diagnosis cannot be obtained without considering a number of differential diagnoses, requiring further biochemical analysis and tissue sampling for histological study. 

## References

[1] Lee JH, Kim K, Chung SW (2001). A case report of inflammatory peudotumor involving the clivus: CT and MR findings. *Korean Journal of Radiology*.

[2] De Vuysere S, Hermans R, Sciot R (1999). Extraorbital inflammatory pseudotumor of the head and neck: CT and MR findings in three patients. *American Journal of Neuroradiology*.

[3] Narla LD, Newman B, Spottswood SS (2003). Inflammatory pseudotumor. *Radiographics*.

[4] Lee EJ, Jung SL, Kim BS (2005). MR imaging of orbital inflammatory pseudotumors with extraorbital extension. *Korean Journal of Radiology*.

[5] Bramwit M, Kalina P, Rustia-Villa M (1997). Inflammatory pseudotumor of the choroid plexus. *American Journal of Neuroradiology*.

[6] Mahr MA, Salomao DR, Garrity JA (2004). Inflammatory orbital pseudotumor with extension beyond the orbit. *American Journal of Ophthalmology*.

[7] McKinney AM, Short J, Lucato L (2006). Inflammatory myofibroblastic tumor of the orbit with associated enhancement of the meninges and multiple cranial nerves. *American Journal of Neuroradiology*.

